# Supplementary material for: Curated and harmonised transcriptomics datasets of interstitial lung diseases
Source: Data Brief. 2025 Oct 14;63:112139. doi: 10.1016/j.dib.2025.112139 (PMC12581653; doi:10.1016/j.dib.2025.112139)
Supplement: Supplementary file 1 [file mmc1.zip › Supplementary_material/RNA-seq/fastQC reports/GSE184316/TRIMMED_SRR15926746_fastqc.html]

TRIMMED\_SRR15926746.fastq.gz FastQC Report 

FastQC Report

Sat 3 Dec 2022  
TRIMMED\_SRR15926746.fastq.gz

## Summary

- Basic Statistics
- Per base sequence quality
- Per sequence quality scores
- Per base sequence content
- Per sequence GC content
- Per base N content
- Sequence Length Distribution
- Sequence Duplication Levels
- Overrepresented sequences
- Adapter Content

## Basic Statistics

| Measure | Value |
| --- | --- |
| Filename | TRIMMED\_SRR15926746.fastq.gz |
| File type | Conventional base calls |
| Encoding | Sanger / Illumina 1.9 |
| Total Sequences | 8116355 |
| Sequences flagged as poor quality | 0 |
| Sequence length | 35-372 |
| %GC | 49 |

## Per base sequence quality

## Per sequence quality scores

## Per base sequence content

## Per sequence GC content

## Per base N content

## Sequence Length Distribution

## Sequence Duplication Levels

## Overrepresented sequences

| Sequence | Count | Percentage | Possible Source |
| --- | --- | --- | --- |
| GGAAGACAGTGTGGCGATTCCTCAGGGATCTAGAACTAGAAATACCATTG | 145121 | 1.7880070548910192 | No Hit |
| ATTGATTCTTCCTACCCATGAGCATGGAATGTTCTTCCATTTGTTTGTAT | 106464 | 1.3117218258688783 | No Hit |
| GAAGGACCTCTTCAAGGAGAACTACAAACCACTGCTCAAGGAAATAAAAG | 101551 | 1.251189727408424 | No Hit |
| ATAGCAGCATGATTTATAGTCCTTTGGGTATATACCCAGTAATGGGATGG | 92971 | 1.1454772493317504 | No Hit |
| GAAGGACCTCTTCAAGGAGAACTACAAACCACTGCTCAATGAAATAAAAG | 69118 | 0.8515891677976136 | No Hit |
| ATAGCAGCATGATTTATAATCCTTTGGGTATATACCCAGTAATGGGATGG | 45562 | 0.5613603643507461 | No Hit |
| ATTGATTCTTCCTACCCATGAGCATGGAATGTTCTTCCATTTGTTTGTGT | 25910 | 0.3192319705089292 | No Hit |
| GGAAGACAGTGTGGCGATTCCTCAAGGATCTAGAACTAGAAATACCATTG | 23657 | 0.2914732044125719 | No Hit |
| GGCCATTTTCATGATATTGATTCTTCCTACCCATGAGCATGGAATGTTCT | 21737 | 0.26781726526254707 | No Hit |
| GCATGTGTCTTTATAGCAGCATGATTTATAGTCCTTTGGGTATATACCCA | 21129 | 0.2603262178650392 | No Hit |
| GGAAGACAGTGTGGTGATTCCTCAGGGATCTAGAACTAGAAATACCATTG | 19615 | 0.24167252418111335 | No Hit |
| ATAGCAGCATGATTTATAGTCATTTGGGTATATACCCAGTAATGGGATGG | 17991 | 0.2216635423167173 | No Hit |
| GGAAGACAGTGTGGCGATTCCTCAGGGATCTAGAACTAGAAATACCATTT | 17704 | 0.21812747224585421 | No Hit |
| GGAAGACAGTGTGGCGATTCCTCAGGGATCTAGAACTGGAAATACCATTG | 16312 | 0.2009769163620862 | No Hit |
| GGAAGACAGTGTGGCAATTCCTCAGGGATCTAGAACTAGAAATACCATTG | 15788 | 0.1945208163023919 | No Hit |
| CACCTCCCCGAAGCTCAGGGAGAGCCCTGTTAGGGCCGCCTCTGGCCCTA | 13862 | 0.1707909523425232 | No Hit |
| ATTGATTCTTCCTATCCATGAGCATGGAATGTTCTTCCATTTGTTTGTGT | 13782 | 0.1698052882112722 | No Hit |
| CAGTGGGGCCACGAGCTGAGTGCGTCCTGTCACTCCACTCCCATGTCCCT | 13492 | 0.1662322557354872 | No Hit |
| AGGCTACACACTGAGGACTCTGTTCCTCCCCTTTCCGCCTAGGGGAAAGT | 13090 | 0.1612792934759507 | No Hit |
| GAAGGACCTCTTCAAGGAGAACTACAAACCACTGCTCAACGAAATAAAAG | 13050 | 0.1607864614103252 | No Hit |
| GGAAGACAGTGTGGTGATTCCTCAAGGATCTAGAACTAGAAATACCATTG | 12671 | 0.1561168775885234 | No Hit |
| GCATGTGTCTTTATAGCAGCATGATTTATAATCCTTTGGGTATATACCCA | 12398 | 0.15275329874062926 | No Hit |
| GGAAGACAGTGTGGCAATTCCTCAAGGATCTAGAACTAGAAATACCATTG | 11264 | 0.13878150968014583 | No Hit |
| ATTGATTCTTCCTACCCATGAGCATGGAATGTTCTTCCATTTCTTTGTAT | 10467 | 0.1289618307725574 | No Hit |
| TGATATGGTTCCTGGCAAGCCCATGTGTGTTGAGAGCTTCTCAGACTATC | 10113 | 0.12460026699177154 | No Hit |
| CAATCCACTTGATCCCAACTCATCTCTCATTTATTTCGGCTTCTTTTATT | 9856 | 0.1214338209701276 | No Hit |
| GGGAAGCGGGGAATGTCTACGTGCGTATGCACGTGGCACTCTCTGCCCGA | 9688 | 0.11936392629450043 | No Hit |
| TGATGACACCCACCGCAACTGTCTGTCTCATATCACGAACAGCAAAGCGA | 9299 | 0.11457113445629227 | No Hit |
| CCAACTCGGGGGCCTGGAGGCAGGGGTGGTCTCTCTTGAGCTCTCCTTGC | 9064 | 0.11167574607074235 | No Hit |
| TTACAAAAGCAGAATAAAAGCGAAAAGAAATGAAAATGTTACACTACATT | 8739 | 0.10767148553753501 | No Hit |

## Adapter Content

Produced by FastQC (version 0.11.7)
